# Evolutionary divergence of core and post-translational circadian clock genes in the pitcher-plant mosquito, *Wyeomyia smithii*

**DOI:** 10.1186/s12864-015-1937-y

**Published:** 2015-10-06

**Authors:** Duncan Tormey, John K. Colbourne, Keithanne Mockaitis, Jeong-Hyeon Choi, Jacqueline Lopez, Joshua Burkhart, William Bradshaw, Christina Holzapfel

**Affiliations:** Institute of Ecology and Evolution, University of Oregon, Eugene, OR USA; Stowers Institute for Medical Research, Kansas City, MO USA; Center for Genomics and Bioinformatics, Indiana University, Bloomington, IN USA; School of Biosciences, University of Birmingham, Birmingham, UK; Pervasive Technology Institute, Indiana University, Bloomington, IN USA; GRU Cancer Center, Georgia Regents University, Augusta, GA USA; Department of Biological Sciences, Notre Dame University, Notre Dame, IN USA; Burke E. Porter Machinery, Grand Rapids, MI USA

**Keywords:** Transcriptome, Transcription-translation feedback, Post-translational modifiers, Gene sequencing, Gene alignment, Biological clocks, Culicidae, Sabethini

## Abstract

**Background:**

Internal circadian (*circa*, about; *dies*, day) clocks enable organisms to maintain adaptive timing of their daily behavioral activities and physiological functions. Eukaryotic clocks consist of core transcription-translation feedback loops that generate a cycle and post-translational modifiers that maintain that cycle at about 24 h. We use the pitcher-plant mosquito, *Wyeomyia smithii* (subfamily Culicini, tribe Sabethini), to test whether evolutionary divergence of the circadian clock genes in this species, relative to other insects, has involved primarily genes in the core feedback loops or the post-translational modifiers. Heretofore, there is no reference transcriptome or genome sequence for any mosquito in the tribe Sabethini, which includes over 375 mainly circumtropical species.

**Methods:**

We sequenced, assembled and annotated the transcriptome of *W. smithii* containing nearly 95 % of conserved single-copy orthologs in animal genomes. We used the translated contigs and singletons to determine the average rates of circadian clock-gene divergence in *W. smithii* relative to three other mosquito genera, to *Drosophila*, to the butterfly, *Danaus*, and to the wasp, *Nasonia*.

**Results:**

Over 1.08 million cDNA sequence reads were obtained consisting of 432.5 million nucleotides. Their assembly produced 25,904 contigs and 54,418 singletons of which 62 % and 28 % are annotated as protein-coding genes, respectively, sharing homology with other animal proteomes.

**Discussion:**

The *W. smithii* transcriptome includes all nine circadian transcription-translation feedback-loop genes and all eight post-translational modifier genes we sought to identify (Fig. 1). After aligning translated *W. smithii* contigs and singletons from this transcriptome with other insects, we determined that there was no significant difference in the average divergence of *W. smithii* from the six other taxa between the core feedback-loop genes and post-translational modifiers.

**Conclusions:**

The characterized transcriptome is sufficiently complete and of sufficient quality to have uncovered all of the insect circadian clock genes we sought to identify (Fig. 1). Relative divergence does not differ between core feedback-loop genes and post-translational modifiers of those genes in a Sabethine species (*W. smithii*) that has experienced a continual northward dispersal into temperate regions of progressively longer summer day lengths as compared with six other insect taxa. An associated microarray platform derived from this work will enable the investigation of functional genomics of circadian rhythmicity, photoperiodic time measurement, and diapause along a photic and seasonal geographic gradient.

**Electronic supplementary material:**

The online version of this article (doi:10.1186/s12864-015-1937-y) contains supplementary material, which is available to authorized users.

## Background

The rotation of the earth about its axis generates a daily cycle of light, temperature, moisture and resources that ultimately affect the microclimate and fitness of organisms [1–5]. A general property of Eukaryotes is that they possess an internal, self-sustaining circadian (*circa*, about; *dies*, day) clock that results in the anticipation and preparation for daily changes in both their external and internal environments [6–9]. Circadian rhythms “are inherent in and pervade the living system to the extent that they are fundamental features of its organization; and to an extent that if deranged, they impair it” ([6], p. 159). Indeed, studies from prokaryotes to mammals have shown that impairment of the circadian clock or imposition of daily environmental cycles that deviate from the innate duration or period of the circadian clock results in reduced fitness [6, 7, 10]. Even if the period of the clock is exactly 24 h, the clock will be able to track the daily cycle of light and dark if the oscillator driving the rhythm varies in its responsiveness to light through the daily cycle [11, 12]. Hence, life in a 24-h world should impose stabilizing selection for a biological clock with an innate period of about 24 h.

**Fig. 1:**
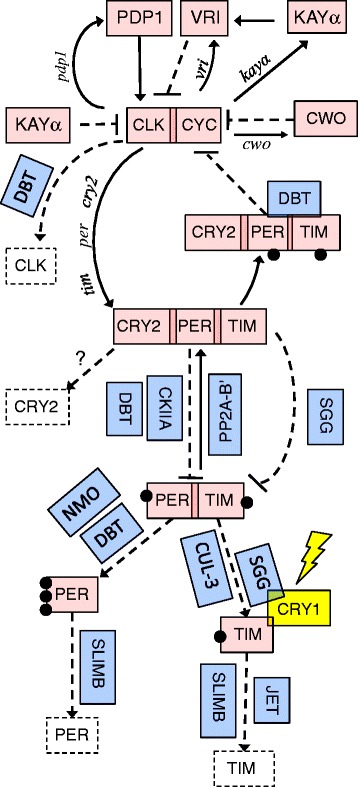
Functional clockworks of the genes listed in Table 2. Pink: TTFL genes, the core transcription-translation feedback loop consists of positive-acting CLK and CYC and negative-acting CRY2, PER, and TIM; their cycling is affected by “stabilizing” loops involving CWO, KAYα, VRI, and PDP1. Blue: PTM genes, the duration of the circadian cycle is then altered by a number of post-translational modifiers, mainly kinases and phosphatases. Yellow: Entrainment of the circadian clock by external day and night is achieved via the blue-light receptor CRY1. Clear dashed boxes: phosphorylation or ubiquitination leading to ultimate protein degradation. Solid arrows: enhancing transcription or PP2A-B’ reversing phosphorylation of PER. Dashed lines: inhibiting transcription or promoting phosphorylation. Upper case Roman, proteins; lower case Italic, transcripts promoted by CLK and CYC. Solid black circles: phosphate groups (compiled from [17, 25, 26, 30, 123, 125, 127])

At the core of all eukaryotic circadian clocks are transcriptional-translational feedback loops (TTFL, pink in Fig. 1) [13, 14]. The concept of the TTFL existed before any clock genes were known [15] and has been described as comprising the “core” or canonical clock genes. Very quickly, it was recognized in *Drosophila* that the TTFL consisted of positive-acting elements (CLK/CYC) and negative-acting elements (PER/TIM) with input of light through CRY1 (aka dCRY) and its interaction with TIM and SGG. Subsequently, the PDP1, VRI, KAYα, and CWO feedback loops have been shown to interact with and regulate transcription in the CLK/CYC – PER/TIM cycle. We also included in our analyses CRY2 (aka mCRY) because, unlike in *Drosophila*, it is known to be a transcriptional regulator of TTFL genes in mosquitoes, Lepidoptera, Hemiptera, Orthoptera, and Hymenoptera, as well as mice [16–20].

Straightforward kinetics of the TTFL estimate that, unmodified, the TTFL would complete its cycle in a few hours [14, 21–24] and therefore be poor at orchestrating daily events. This observation elevated the appreciation of post-translational modifiers (PTM, blue in Fig. 1) that act as modulators (governors), delaying this cycle and thereby producing a rhythm of about 24 h [21–36]. It is the quality and quantity of phosphorylation by the PTMs that determine the kinetics of the negative-acting loop and, hence, the period of the circadian clock and ultimate degradation of the TTFL proteins [21, 24, 31–36]. Hence, it has been proposed that the post-translational or the post-transcriptional modifiers are more responsible for maintenance of a biological clock with a period of about 24 h than is the TTFL [21–23, 36–42]. This proposition would predict that PTM genes should be evolutionarily more conservative than TTFL genes.

Herein, we investigate the relative evolutionary rates of TTFL and PTM genes using the mosquito *Wyeomyia smithii*. The roles of post-transcriptional control [37, 40–42], micro-RNAs [38, 39, 43], *O*-GlcNAcylation [44], and histone acetylation and methylation [22, 45, 46] in circadian time-keeping are still emerging areas of research, especially in insects. Hence, We focused on the TTFL and phosphorylation-related PTM as the two best documented groups of genes involved in circadian rhythmicity that possessed both distinct roles in the circadian clock (TTFL vs. PTM) and distinct biochemical mechanisms (regulation of gene transcription vs. modification of protein stability).

The genus *Wyeomyia* is a member of the tribe Sabethini, which includes some 429 circumtropical species [47], only one of which, *W. smithii*, has invaded temperate North America, likely from tropical South America [48]. *Wyeomyia smithii* completes its pre-adult development only in the water-filled leaves of the carnivorous plant *Sarracenia purpurea* and has dispersed northwards from the Gulf of Mexico to northern and western Canada [49–51]. Over a similar south to north geographic range, the oviposition rhythm of *Drosophila melanogaster* has shown a decline in amplitude, and the eclosion rhythms of *D. subobscura* and *D. littoralis* have shown a decline in both amplitude and period [52]. This latitudinal gradient in period and amplitude of the circadian clock has been attributed to summer day length, which increases with latitude, thereby imposing selection for an increasingly robust oscillator, although evidence supporting this proposition remains equivocal [52, 53]. Regardless of the ultimate causality of the latitudinal gradient in *Drosophila*, *W. smithii* has encountered the same gradient in summer day lengths and we ask whether there has been greater rates of divergence in PTM or TTFL genes in a northern, derived population of *W. smithii* relative to other insects. We focus on a northern population of *W. smithii* first because we were able to use the recently collected F2 of field-collected larvae that reflect the genomics of a natural population. Second, we have over 30 years experience working with the genetics, evolution, physiology, and population biology of *W. smithii* from the Gulf of Mexico to northern Canada, including this particular population (http://www.uoregon.edu/~mosquito). We are therefore able to place our ongoing genomics experiments into a broader context relating to the bionomics of the focal species. Finally, this population represents a more polar population than any other Sabethine mosquito; the only other temperate Sabethine (*Trypteroides bambusa*) occurs in East Asia and does not reach the latitude (46 °N) of the focal population [54]. Hence, this population represents a more northern and, therefore, is more likely to parallel *Drosophila* in the northern, post-glacial divergence of its circadian clock than any other Sabethine species.

At present, there are no sequenced genomes or transcriptomes available for any member of the circumtropical mosquito tribe Sabethini, among which several Neotropical species, including members of the genus *Wyeomyia*, but not including *W. smithii*, have been implicated in the transmission of arboviruses [55, 56]. We therefore produced the first Sabethine transcriptome sequence, assembly and gene annotation. We compared amino acid substitutions from translated *W. smithii* sequences with annotated circadian clock genes in other insects and compared the sequence divergence between *W. smithii* and six other taxa of increasing phylogenetic divergence : mosquitoes in the same subfamily but different tribes (*Aedes* and *Culex*), a mosquito in a different subfamily (*Anopheles*), another Diptera in a different sub-order (*Drosophila*), and progressively more distant orders (Lepidoptera, *Danaus*; Hymenoptera, *Nasonia*). We compared evolutionary rates using nine genes of the TTFL with eight key genes of the PTM (Fig. 1). All six species we considered exhibit circadian rhythmicity under daily and constant conditions [16, 25, 27, 52, 57–60]. Finally, we estimated evolutionary divergence from branch lengths of the generated maximum-likelihood tree for each gene. Our goal was to present the Sabethine transcriptome, a concise application of that transcriptome, and to emphasize concepts rather than present a discussion of the genome-wide details of the transcriptome.

We made four basic assumptions: First, during its dispersal northwards in North America, *W. smithii* has undergone analogous directional selection on its circadian clock as reflected in circadian-based behaviors in *Drosophila melanogaster, D. subobscura, and D. littoralis*. Second, directional selection and drift will erode genetic variation in clock genes as it has in other protein-coding loci in *W. smithii* [50]; consequently, genes under stronger selection will exhibit, on average, shorter branch lengths between this northern population of *W. smithii* and the other taxa. Third, the sequence reads from the *W. smithii* transcriptome represent random samples of their respective genes. This third assumption bears the caveat that, from incomplete cDNA contigs in the assembly, we cannot estimate evolutionary rates of individual genes, since different domains and even different codons within a domain, may evolve at different rates [31]. Since we are aligning *W. smithii* sequences of varying completeness to identify orthologs across disparate taxa, there is an inherent bias towards enriching for more conserved segments of the clock genes. Since we are concerned with the comparative evolutionary rates of functional groups of genes in taxa that are separated by 100-400my, this temporal separation means that we have to use more conservative portions of the genes involved in order to obtain a clear signal of protein divergence. Nonetheless, if conservative segments are randomly distributed among clock genes, average divergence of TTFL or PTM genes provides a composite estimate of those two functional components of the *W. smithii* circadian clock. Fourth, we assume that TTFL and PTM genes identified in *Drosophila* serve analogous functions in the other insect taxa we consider. The number and function of circadian clock genes is better documented in *Drosophila*, which has set the historical landmarks for comparison with other insects and mammals [12, 14, 21, 22, 61–63] When looked for, the TTFL genes that are rhythmically expressed in *Drosophila* are also found to be rhythmically expressed in *Danaus* [18] and *Nasonia* [16] as well as mosquitoes [58–60, 64, 65] (including *tim* in *W. smithii* [66]). Functionally, RNAi targeted against *Cry2* [16–19], *tim* [64, 67–69], *per* [70–72], *Clk* [73, 74], *cyc* [75, 76] all disrupted circadian rhythmicity in non-*Drosophila* insects ranging from other Diptera to apterygote Thysanura. At least *Cry2* and TTFL orthologs of *tim*, *per*, *Clk*, and *cyc* in *Drosophila* are involved in circadian clock function across a variety of insects.

## Methods

### Collection, maintenance, and experimental treatment of *Wyeomyia smithii*

*Wyeomyia smithii* were collected in spring, 2010, as overwintering larvae from Maine (46 °N, 68 °W, 270 m elevation; population KC of earlier studies from this lab). Populations were maintained at the University of Oregon under standard rearing conditions and run through two generations to minimize maternal and field effects [53]. In the F2 laboratory generation, larvae were reared on short days (L:D = 8:16) at 23 °C to induce larval diapause in the third instar. After the initiation of diapause, a group of larvae continued on short days while another group was directly transferred to long days (L:D = 18:6) in order to initiate development, both at 23 °C.

### RNA isolation and cDNA library construction, transcriptome sequencing and assembly

RNA was extracted from 12 samples of 30 individuals each. The 12 samples represented diapausing larvae on short days (L:D = 8:16), diapausing larvae exposed to 10 diapause-terminating long days (L:D = 18:6), pupae on long days and adults on long days. Each stage of development was sampled at three times of day (Table 1). All samples were prepared in 500uL TRIzol (Ambion Life Technologies, 5791 Van Allen Way, Carlsbad, California 92008) according to manufacturer’s protocol. RNA was resuspended in 20uL DEPC-treated water and stored at -70 °C until shipment on dry ice to the Center for Genomics and Bioinformatics at Indiana University.

**Table 1:** Equimolar sources of cDNA to generate the *W. smithii* transcriptome

Sample	Stage	Daylength^a^	Hours after lights-on
1	larvae	Short	5
2	larvae	Short	13
3	larvae	Short	21
4	larvae	Long	5
5	larvae	Long	13
6	larvae	Long	21
7	pupae	Long	5
8	pupae	Long	13
9	pupae	Long	21
10	adult	Long	5
11	adult	Long	13
12	adult	Long	21

^a^Short: L:D = 8:16; long: L:D = 18:6

The overall quality of RNA samples was evaluated in terms of purity and integrity of RNA by means of a NanoDrop ND-1000 UV–VIS spectrometer (Thermo Fisher Scientific, 81 Wyman St, Waltham, MA 02451), Bioanalyzer (Agilent Technologies, 5301 Stevens Creek Blvd., Santa Clara, CA 95051) and agarose gel electrophoresis. RNA sample quality was verified regarding high RNA concentration, absorbance ratios A260/A280 in the range 2.0 - 2.2, and A260/A230 above 1.8. Samples with lower absorbance ratio were ethanol-precipitated in order to improve the quality. Equivalent amounts of RNA mass per test condition were pooled together, with a total of 10 μg RNA from all samples of *W. smithii*. Normalized 454-sequencing libraries were constructed from an equal-molar pool of RNA obtained from the unique exposure samples described above using the procedures optimized for Roche/454 Titanium sequencing modified from Meyer et al. [77]. After the final purification step, the library was stored at −20 °C until sequencing. This library was sequenced using one full-plate sequencing run in a 454 Roche GS FLX pyrosequencing instrument with Titanium chemistry (454 Life Sciences Corporation, 15 Commercial St., Branford, CT 06405), following manufacturer’s protocol and methods previously described [78]. After 454 sequencing, the generated sequence reads were cleaned using ESTclean [79] and assembled using Newbler v.2.5.3 (454 Life Sciences Corporation, 15 Commercial St., Branford, CT 06405) in *de novo* mode and default parameters.

### Transcriptome annotation

Transcriptome annotation was performed through the ISGA transcriptome analysis pipeline [80]. First, sequence homology to known metazoan proteins was obtained by submitting contigs and singletons to BLASTx searches against NCBI’s non-redundant database and dbEST [81]. Moreover, protein domains were identified among the six frame translations of the assembled sequences using Pfam, TIGRfam and HMMER3 searches [82]. Open reading frames were determined with the ORFpredictor software on the proteomics server of Youngstown State University [83]. Finally, orthology and paralogy was assigned by BLASTx against orthoMCL databases [84].

### Defining orthologous groups and ortholog sequence acquisition

**Fig. 2:**
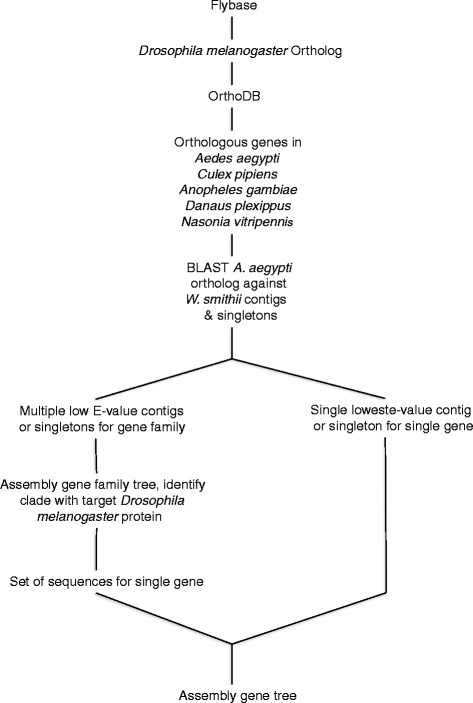
Flow diagram of assigning contigs or singletons to specific circadian clock genes. The functional circadian clock gene was identified in *Drosophila melanogaster* through Flybase. The *Drosophila melanogaster* protein sequence was blasted against OrthoDB7 using the most recent common ancestor of all seven species as the search node. The orthologous genes were then taken from the resulting OrthoDB group, with the ortholog of *A. aegypti, W. smithii*’s most closely related species, and used in a local BLAST against the contigs and singletons from the *W. smithii* transcriptome. If the lowest E-value from that BLAST identified a single contig or singleton, that contig or singleton was assigned to the respective *D. melanogaster* gene function in the OrthoDB group. If the lowest E-value from the BLAST identified a multi-gene family, maximum likelihood trees were used to identify the orthologs of various genes in that family (Figs. 3 and 4)

**Fig. 3:**
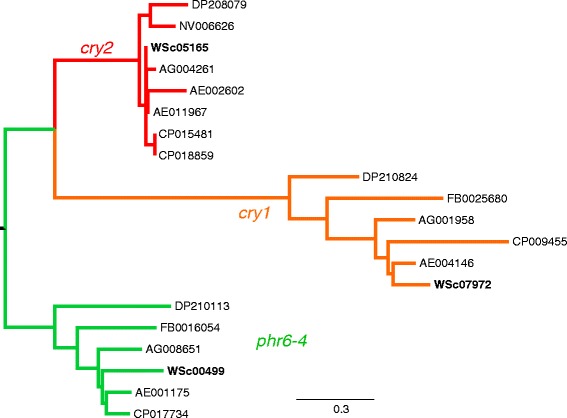
Assigning *W. smithii* orthologs to *cry1*, *cry2*, and *phr6-4* (*6*–*4 photolyase*). The maximum likelihood tree identified a single *W. smithii* contig (bold) within each of the three monophyletic clades in the tree. Gene number abbreviations: AE, *Aedes aegypti*; AG, *Anopheles gambiae*; CP, *Culex pipiens*; DP, *Danaus plexippus*; FB, *Drosophila melanogaster*; NV, *Nasonia vitripennis*; WSc, *W. smithii* contigs

**Fig. 4:**
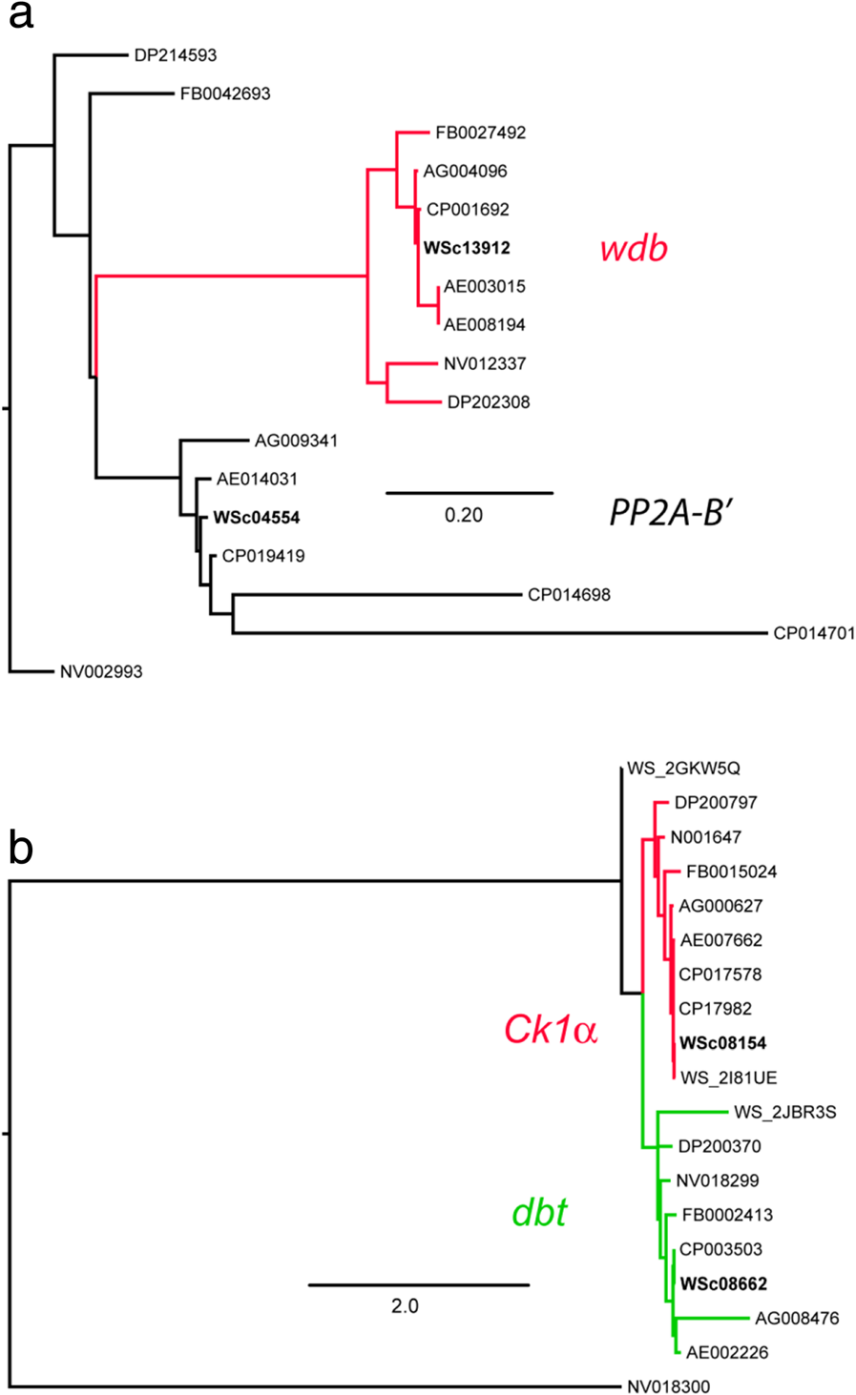
Assigning *W. smithii* orthologs of (**a**) *PP2A-B’* and *widerborst* (*wdb*) and (**b**) *Casein kinase 1α* (*Ck1α*) and *doubltime* (*dbt*). In **a**, *wdb* emerges as a clade within *PP2A-B’* and the *W. smithii* Contig WSc04554 was assigned to *PP2A-B’*. In **b**, *W. smithii* Contigs WSc08154 and WSc08862 (bold) were assigned to *Ck1α* and *dbt*, respectively. Gene number abbreviations as in Fig. 3

Flybase was used to identify each individual circadian gene in *Drosophila melanogaster* [85]. The *D. melanogaster* Flybase gene numbers and peptide sequences were used to identify the pre-computed orthologous genes for all insects using OrthoDB7 [86] and specifically extracting amino acid sequences for five comparative species: *Aedes aegypti, Culex pipiens, Anopheles gambiae, Danaus plexippus* and *Nasonia vitripennis* (Fig. 2). Geneious [87] was used to perform a local BLAST (tblastx) of each *Aedes aegypti* ortholog against the entire *W. smithii* transcriptome. This procedure identified all possible homologous genes as contigs/singletons coding for *W. smithii* clock proteins, except for five groups of related genes that required additional analysis (Table 2): *Clk vs. cyc, tim vs. tim2* (*timeout*), *cry1 vs. cry2 vs. phr6-4*, and *dbt vs. Ck1α*. The contigs/singletons were then each evaluated as representing full gene transcripts, partial gene transcripts (including split genes), orthologs or paralogs, based on the alignments of their translated amino acids to those from the other six species, including their relative positions within the resulting phylogenetic gene trees (Figs. 3 and 4; Additional file 1 and Additional file 2).

**Table 2:** Circadian clock gene acronyms, names, and function

Abbreviation	Gene name	Clock function (See Fig. 1)	Reference
*CkIα*	*casein kinase Iα*	paralog of *dbt*	Fig. 4b
*CkIIα*	*casein kinase IIα*	promotes nuclear localization of PER-DBT-TIM via phosphorylation of TIM	[25–27]
*Clk*	*Clock*	transcription factor promoting transcription of *per, tim, cry2, vri, PDP1, cwo*	[25–27, 118]
*Cul3*	*Cullin 3*	ubiquitinates TIM leading to its degradation	[30]
*cwo*	*clockwork orange*	acts synergisticly with PER to inhibit CLK-mediated gene activation	[25, 119, 120]
*cry*	*cryptochrome 1, cry1, dcry*	photoreceptor; complexes with SGG & TIM to mediate light-input pathway into the clock	[25, 26]
*cry2*	*cryptochrome 2, mcry*	transcription regulator of *Clk* & *cyc * in insects other than Drosophila	[25, 27]
*cyc*	*cycle*	transcription factor promoting transcription of per, tim, cry2, *Pdp1*, vri	[25–27]
*dbt*	*doubltime = dco, discs overgrown*	major regulator of PER & CLK through phosphorylation	[25–27, 31]
*jet*	*jetlag*	promotes light-induced proteosomal degradation of TIM & CRY1	[27, 33, 121]
*kayα*	*kayakα*	Inhibits VRI suppression of *Clk* promoter; represses CLK activity	[127]
*nmo*	*nemo*	phosphorylates PER, enhances action of DBT; phosphorylates CLK	[25, 29, 31, 123]
*Pdp1*	*PAR-domain protein 1*	Likely in combination with VRI enhances *Clk* transcription and clk mRNA amplitude	[25–27, 118]
*per*	*period*	negative transcription regulator of *Clk* & *cyc* transcription after transport to nucleus as phosphorylated PER-TIM-DBT complex	[25, 27]
*phr6-4*	*(6–4)-photolyase*	paralog of *cry1 *&* cry2*	[17], Fig. 3
*PP2A-B’*	*protein phosphatase 2A, regulatory B subunit*	regulates phosphorylation of PER; counter-balances PER & TIM phosphorylation by DBT	[25–27, 31]
*sgg*	*shaggy*	phosphorylates TIM, in concert with CRY1 & light; regulates PER phosphorylation-dephosphorylation; promotes PER nuclear localization	[25–27, 31]
*slimb*	*supernumerary limbs*	phosphorylates PER & TIM, leading to their degradation	[25–27, 124]
*tim*	*timeless*	binds to & facilitates transport of heterodimeric PER into the nucleus; interacts with JET, SGG & CRY to regulate the input of light; increases CKIIα phosphorylation of PER	[25, 125]
*tim2*	*timeout*	paralog of TIM; necessary for laval survivorshiop; promotes chromosomal stability; enhancs photoreception.	[122, 126]
*vri*	*vrille*	transcription inhibitor of *Clk*	[25–27, 118]

### Alignment processing and gene tree assembly

The orthologous groups of amino acid sequences were gathered into their respective gene families for each clock gene, including the translated *W. smithii* representative sequences. The 5’ and 3’ UTRs of each *W. smithii* amino acid sequence were removed based on start and stop codon positions. Each gene family was then aligned using MUSCLE [88] (Additional file 3) The protein alignments were then subjected to Gblock editing in order to identify conserved regions for phylogenetic analysis [89, 90] (Additional file 4 and Additional file 5). In order to be processed by ProtTest and Phyml, the alignments were converted into Phylip format. This conversion involved truncation of the identifiers for certain species' sequences. The identifiers were truncated in such a way to preserve the associated gene number, while changing the organism text identifier (Additional file 6). The best fit models of amino acid replacement for the Gblock edited alignments were determined using ProtTest [91, 92]. Maximum likelihood gene trees were then assembled using the phylogenetic software Phyml [93] and the best model of amino acid substitution according to the ProtTest results (Additional file 2).

## Results

### Transcriptome

Quality filtering of the reads was performed before assembly by applying default parameters using methods described by Vera et al. [94]. The assembly was performed on 1,081,284 quality-controlled reads summing up 94 % of raw sequence data (432,542,060 bases), after trimming of adaptor sequences (Table 3). Newbler aligned 92 % and assembled 87 % of quality-controlled reads, resulting in 25,904 contigs with lengths >50 bases, 14,459 contigs with lengths >500 bases, and 54,418 singletons (Table 3). The N50 for contig length >500 bases was 1373 bp. The Newbler assembler considers alternative splicing that resulted in the integration of contigs into 21,233 isotigs representing candidate transcripts. The N50 for isotigs was 1953 bp and the average size for isotigs was 1515 bp long (Table 3).

**Table 3:** Sequencing results and assembly statistics

Sequencing results	Total	Aligned	Assembled
Number of reads^a^	1,081,284	999,150	936,525
Number of bases	432,542,060	401,519,734	380,523,665
Assembly statistics	Singletons	Number of Contigs (Contigs >500 bp)	Isotigs
Number of sequences	54,418	25,904 (14,459)	21,233
Number of bases	20,109,580	19,120,348 (16,952,075)	32,183,643
Average sequence size (bp)	355	738 (1,172)	1,515
Length of N50 sequence (bp)^b^	451	1,249 (1,373)	1,373

^a^After quality filtering steps and removal of outliers such as adaptor sequences and repeats

^b^N50 is a weighted median statistic, such that 50% of all bases are contained in sequences ≥N50 length

Assembly quality was tested by retrieving BLASTx hits against the *Drosophila* orthologs in the CEGMA core eukaryotic genes dataset [95] (Additional file 7). The contigs alone represent 493 of 523 genes known to exist as single copies, indicating that the transcriptome is >94 % complete.

From the total number of contigs and singletons, a significant BLASTx match was obtained for 13,470 (52 %) and 15,048 (28 %) of transcripts respectively (Additional file 8). This result implies that between 48 % (contigs) and 72 % (singletons) of the sequences do not show homology to any other sequence present in the investigated databases. However, among the 47,837 orphan transcripts, 33,183 have identifiable functional protein domains plus an additional 7486 have detectable open reading frames, indicating that they represent protein-coding genes as well as non-coding transcripts.

Gene Ontology (GO) terms were assigned to 10 % of singletons and 42 % of contigs; overall 11,342 sequences were mapped. Finally, orthoMCL [96] and OrthoDB [97] analyses of gene orthology revealed that 12,653 contigs and 11,787 singletons show orthology to one or more organisms in the two gene-orthology databases (Additional file 8).

### Defining orthologous groups

The *Wyeomyia smithii* transcriptome included all 17 circadian clock genes we sought to identify (Fig. 1). The clock genes were represented by 15 contigs and two singletons, ranging from 450 to 3000 nucleotides (Table 4). As expected, *cry2* is absent in *D. melanogaster* and both *tim* and *cry1* are absent in *Nasonia vitripennis* [17, 25, 27, 98, 99]. The local BLAST (tblastx) of each *Aedes aegypti* ortholog against the *W. smithii* transcriptome identified a single best contig or singleton to represent 11 of the clock genes. Six other clock genes belonged to broader gene families and required additional analysis.

**Table 4:** Circadian clock genes, their role in the clock, properties of their *Wyeomyia smithii* transcripts and their relationship to homologs in *Drosophila melangaster* and *Aedes aegypti*

Gene^a^	Transcript^b^	*A. aegypti* AAEL- ^c^	E-value^d^	ProtTest Model	FBgn-	Ortho DB EOG7-^e^	Nucleo- tides	Relative Rate
*CkIIα*	15735	012094	0	Dayhoff+G+F	0264492	4RCF1	451	0.102
***Clk***	***2GK8YT***	**012562**	**0**	**JTT+G+F**	**0023076**	**NSNR1**	**1,865**	**0.679**
***cry2***	**05165**	**002602**	**4E-173**	**LG+G**	**---**	**P64PH**	**634**	**0.298**
*Cul3*	07535	006291 007187	0	LG+G	0261268	TN9FJ	1,771	0.348
***cwo***	**15437**	**010513**	**0**	**LG+G+F**	**0259938**	**XDP58**	**2,313**	**1.792**
***cyc***	**17314**	**002049**	**7 E-86**	**JTT+G**	**0023094**	**7TBTD**	**851**	**0.390**
*dbt*	08662	002226	1E-144	JTT+G	0002413	2CGPS	527	0.866
*jet*	03197	012126	1 E-93	LG+G+F	0031652	NSNRX	1,004	1.572
***kayα***	**13013**	**008953**	**8 E-145**	**LG+G+F**	**0001297**	**06BFN**	**1,162**	**1.625**
*nmo*	17870	004797	0	JTT+G	0011817	JF2MQ	2,130	0.254
***Pdp1***	**02480**	**005255**	**8 E-145**	**LG+I**	**0016694**	**2RZNP**	**3,083**	**0.602**
***per***	***1A331S***	**008141**	**1 E-76**	**JTT+G**	**0003068**	**MHBMZ**	**2,670**	**1.500**
*PP2A-B'*	04554	014031	0	LG+G	0042693	S57VZ	721	0.463
*sgg*	17080	005238	0	Dayhoff + G	0003371	D2S3R	2641	0.455
*slimb*	03120	003371	0	JTT+G	0023423	WHV0D	2,355	0.559
***tim***	**06527**	**006411**	**1 E-61**	**JTT+G+F**	**0014396**	**DG81S**	**2,752**	**0.793**
***vri***	**08372**	**011371**	**7 E-133**	**JTT+I+G+F**	**0173452**	**HXQF6**	**2,465**	**1.234**

^a^Bold font, TTFL; regular font, PTM

^b^Contigs in Roman; singletons in italics, omitting the prefix F5BTJ3O0-

^c^ *Aedes aegypti* BLAST to *W. smithii* transcriptome

^d^E-values from reciprocal BLAST of contig or singleton to *A. aegypti*

^e^Rooted at most-recent common ancestor of Hymenoptera & Diptera

*Clk* vs. *cyc*: *Drosophila melanogaster Clk* and *cyc* are represented in two different EOG7 orthologous groups. Local BLASTS of the *A. aegypti* orthologs of *Clk* (AAEL012562) and *cyc* (AAEL002049) against the *W. smithii* transcriptome identified a *W. smithii* singleton (2GK8YT) and a *W. smithii* contig (Contig 17314), that best represented *clk* and cyc, respectively (Table 4).

*tim* vs. *tim2* (*timeout*): *Drosophila melanogaster Clk* and *cyc* are represented in two different EOG7 orthologous groups. Local BLASTS of the *A. aegypti* orthologs of *tim* (AAEL006411) and *tim2* (AAEL009518) against the *W. smithii* transcriptome identified two distinct *W. smithii* contigs that distinguished *tim* (Contig 06527) from *tim2* (Contig 18589), respectively (Tables 4 and 5).

**Table 5:** Genes closely related to clock genes or in the same gene family^a^

Related gene	*W. smithii* Contig	*A. aegypti* AAEL-	E-value	Clock gene	FBgn	Ortho DB EOG7-	Nucleotides
*CkIα*	08154	007662	0	*dco*	0015024	2CGPS	483
*cry1*	07972	004146	0	*cry2, phr6-4*	0025680	9SRM2	1,228
*phr6-4*	00499	001175	0	*cry1, cry2*	0016054	P64PH	1,134
*tgo*	06945	010343	0	*cyc*	0264075	Q2ZVC	940
*tim2*	18589	009518	7 E-45	*tim*	0038118	2RZMT	589
*wbt*	13912	003015 008194	1 E-165	*PP2A-B'*	0027492	S57VZ	4,552

^a^Column headings as in Table 4

*cry1* vs. *cry2* vs. *phr6-4*: Protein sequences belonging to the cryptochrome family were identified and combined from the *cry1, cry2,* and *phr6-4* orthologous groups, EOG79SRM2 and EOG7P64PH. Local BLASTS of the *A. aegypti* sequences from the two OrthoDB groups against the *W. smithii* transcriptome identified three *W. smithii* candidate contigs. After trimming the 5’ and 3’ UTRs, the contigs were Gblock edited, and, in combination with the other six species, tested for the appropriate amino acid substitution model (see Methods). A maximum likelihood tree rooted with *phr6-4* [17, 98, 100] separated sequences into three distinct clades representing *cry1*, *cry2*, and *phr6-4* (Fig. 3). The three *W. smithii* candidate contigs were each placed in a separate clade. We therefore concluded that Contig 07972 is the ortholog of *cry1*, Contig 05165 is the ortholog of *cry2*, and Contig 00499 is the ortholog of *phr6-4*.

*dbt* vs.*Ck1α*: The OrthoDB group for *D. melanogaster doubletime* (EOG72CGPS) contained two gene families, *doubletime* (*discs overgrown*) and *Casein kinase 1α*. A gene tree was then assembled using the same protocols described for the cryptochromes, above. When rooted with the *N. vitripenni*s sequence NV018300, the remaining orthologs separated into two distinct clades one including *D. melanogaster dbt* and *W. smithii* Contig 08662, the other one including *D. melanogaster Ck1α* and *W. smithii* Contig 08154 sequence (Fig. 4a). We therefore concluded that Contig 08662 is the ortholog of *dbt* and Contig 08154 is the ortholog of *Ck1α.*

*PP2A-B’* vs*. wdb*: The OrthoDB group for the *D. melanogaster* ortholog of *PP2A-B’* (EOG7S57VZ) contained two gene families, *PP2A-B’* and *widerborst*. To distinguish these genes in *W. smithii*, the three *A. aegypti* sequences in the same OrthoDB group, were aligned locally against the *W. smithii* transcriptome using BLAST. A gene tree was assembled using the same protocols described for *cry2* showing that the *widerborst* gene family occupied its own monophyletic clade within the *PP2A-B’* gene tree (Fig. 4b). The *wdb* clade included *W. smithii* Contig 13912. A separate branch included *D. melanogaster PP2A-B’* and *W. smithii* Contig 04554. We therefore concluded that Contig 13912 is the ortholog of *wdb*, and Contig 04554 the ortholog of *PP2A-B’*.

### Evolutionary divergence

Divergence of *W. smithii* genes involved in the circadian clock was determined from relative, cumulative branch lengths from other taxa (Table 4) using maximum likelihood for phylogenetic inference. Among the 17 circadian genes (Fig. 1) ProtTest returned six different best-fit models for amino acid substitution for nine TTFL genes and five different models for eight PTM genes (Table 4). The frequency of different models did not differ between the two categories of genes (two-sided Fisher’s exact test *P* = 1.000).

A distance matrix (Additional file 1) was generated for each maximum likelihood gene tree, showing the relative branch lengths between each organism for each particular protein. In order to measure rates of evolution for *W. smithii’s* clock proteins relative to the other organisms in each gene family, relative rate for each clock protein was calculated from the distance matrices:

Relative rate = (Average branch length for *W. smithii* across all taxa for an individual gene) ÷ (average branch length for all seven taxa across all genes). For a given protein, when this ratio is greater than 1.0, it indicates that the protein is evolving faster in *W. smithii* relative to other organisms; when this ratio less than 1.0, it indicates that the protein is evolving more slowly in *W. smithii* relative to other organisms.

**Fig. 5:**
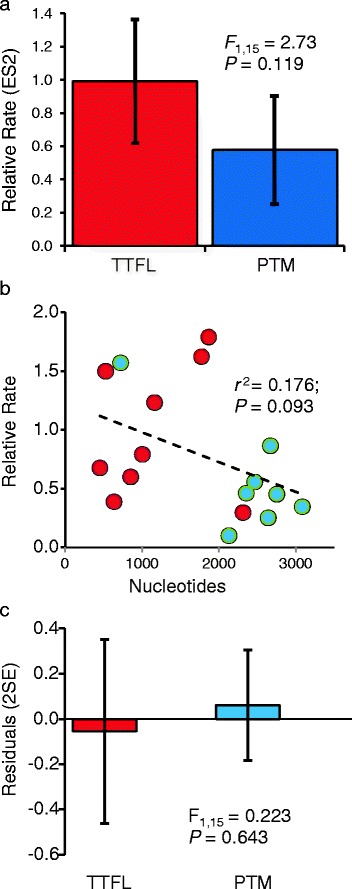
Rates of amino acid divergence in circadian clock genes of *Wyeomyia smithii* relative to other insects (Table 4). **a** Relative rates (±2SE) of divergence in the core transcription-translation feedback loop (TTFL) and of post-transcriptional modifiers (PTM). **b** relationship between relative rates of amino acid divergence and the number of nucleotides in the contigs or singletons upon which the rates were based. TTFL (red) and the PTM (blue). **c** deviations from regression (residuals) in **5b**. The residuals essentially factor out any differences in relative rates due to the number of nucleotides upon which amino acid divergence was based

Relative divergence of *W. smithii* TTFL genes did not differ from 1.0 but divergence of PTM genes was significantly less than 1.0 (Table 6). However, relative divergence of TTFL and PTM genes did not differ significantly from each other (Fig. 5a). There was a marginally non-significant negative correlation between relative rate of combined gene divergence and number of nucleotides in their respective contigs or singletons (Fig. 5b). To account for the possibility of Type II error, ANCOVA of core vs. PTM genes with number of nucleotides as the covariate revealed no significant treatment effect (*t* = 0.621, *P* = 0.545) and ANOVA of the residuals from regression of divergence on number of nucleotides also revealed no significant difference between core and PTM genes (Fig. 5c).

**Table 6:** Relative divergence of W. smithii TTFL and PTM clock genes from other insects

Category^a^	Mean	StErr	*t*	*df*	*P = 1.0* ^b^
TTFL & PTM combined	0.8	0.13	1.55	16	0.14
TTFL only	0.99	0.2	0.49	8	0.96
PTM only	0.58	0.16	2.6	7	0.04

^a^TTFL, transcription-translation feedback loops; PTM: post-translational modifiers

^b^Probability Mean differs from 1.0

## Discussion

Using the “black sheep” counting technique of universal, single copy genes to determine the completeness of the *Wyeomyia smithii* transcriptome, we estimated that the *W. smithii* transcriptome encompasses >94 % of its transcribed genome. This result is not surprising since we used as the basis for the transcriptome both developing and diapausing larvae, pupae, and adults sampled at different times of the day and night and under long and short days (Table 1). In fact, the *W. smithii* transcriptome includes all of the 17 circadian clock genes we sought to identify (Fig. 1); its contigs and singletons translate into peptides of sufficient length to estimate comparative rates of evolution of both TTFL and PTM genes between the *W. smithii* and the six comparison taxa.

**Fig. 6:**
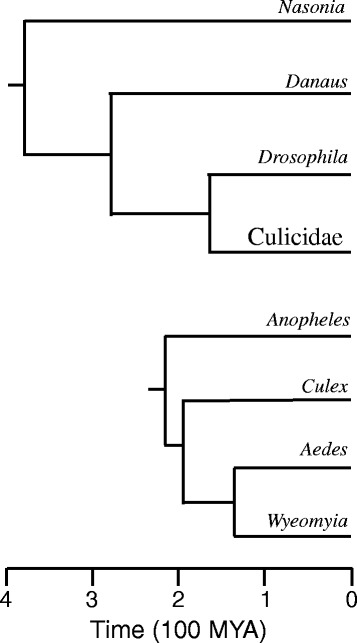
Phylogenetic relationships of insects used in this study. The nodes indicate approximate time since the most recent common ancestor of a given branch. Orders and families (top) based on [128]; genera within the family Culicidae (bottom) based on [129]

Even if historical directional selection on the circadian clock has occurred among populations dispersing along a latitudinal gradient, stabilizing selection at any locality along that gradient is still important in maintaining daily time-keeping in concert with a 24-h world. Concordance between the circadian clock and the external 24-h world is an important component of fitness in organisms from prokaryotes to mammals [6, 7, 10], including *W. smithii* [101]. The motivation for our study was to compare the relative rates of evolutionary divergence of TTFL and PTM genes between a northern population of *W. smithii* that has experienced a continual northward dispersal into temperate regions of progressively longer summer day lengths, with both closely related mosquitoes and more distantly related insects, including *Drosophila melanogaster*, *Danaus plexippus*, and *Nasonia vitripennis* (Fig. 6). Overall, we found that *W. smithii* clock genes are not evolving faster than expected from other insects (Table 6) and the rate of evolution of TTFL genes does not differ from PTM genes (Fig. 5).

The best models for amino acid substitution do not differ between TTFL and PTM genes, although six different models provided the best fit within TTFL genes and five different models within PTM genes, (Table 4). Clearly, neither the TTFL nor the PTM proteins represent a uniform group in terms of their evolution. Consequently, no single substitution model would be appropriate for phylogenetic inference of circadian clock genes within either functional group or within the two groups combined. There is, however, greater retention of PTM than TTFL genes among the six insect taxa we considered. *cry2* is dispensable in *Drosophila* (although present in lower Diptera) and *tim* and *cry1* are dispensable in Hymenoptera [17, 25, 27, 98, 99]. By contrast, all eight of the PTM genes are conserved in all six taxa. This observation indicates that natural selection within and between orders of insects has acted to conserve PTM genes more than TTFL genes.

What importance then are the TTFL genes? To be a functional time-keeper of overt circadian expression, the circadian clockworks cannot work in isolation but must communicate circadian time to downstream clock-controlled genes. “Much is known about how information is relayed to the *Drosophila* [*melanogaster*] clock and how the central clock itself functions, but less is understood about how information from the clock is relayed to the rest of the organism” ([102], p. 352) [103, 104]. Since all of the TTFL genes are transcription factors or transcription regulators of gene expression, it is not surprising that the TTFL genes likely provide this communication to clock-controlled behavioral and physiological processes [37, 104–114]. The TTFL genes provide a cyclical expression of genes and a pleiotropic, time-specific signal to the rest of the organism; the PTMs maintain this cycle with a period of about 24 h. It is the genetic co-adaptation, i.e., the co-evolution within and between these functional groups that enables different organisms to maintain biochemical, physiological, and behavioral activities in concert with the external daily environment.

## Conclusion

We report the first genome or transcriptome of any member of the mosquito tribe Sabethini (subfamily Culicinae). This transcriptome serves as a point of departure for annotating a future scaffolding genome of *W. smithii*. As an application of the transcriptome, we compared rates of evolutionary divergence of *W. smithii* circadian clock genes from six other insect taxa. We found no significant difference in rates of evolutionary divergence between genes involved in the central transcription-translation feedback loop and genes involved in post-translational modifiers. All of the species we considered exhibit circadian rhythmicity under constant conditions and include all the PTM genes in Fig. 1. By contrast, the representation of TTFL genes varies among taxa, including sub-orders of Diptera. This contrast means that there has to be genetic coadaptation both within the TTFLs to maintain a rhythmic circadian output and between the TTFL and their PTMs to maintain that rhythmic output with a period of about 24 h in concert with the 24-h variation in the external environment.

## Additional files

Additional file 1:
**Distance matrices** (XLSX 20 kb) Additional file 2:
**Maximum likelihood trees in Newick format; visualize with FigTree [** 116 **].** (DOC 28 kb) Additional file 3:
**Muscle protein alignments; visualize with MEGA [** 117 **].** (ZIP 46 kb) Additional file 4:
**Gblock edited alignments, FA-GB files in .txt format.** (ZIP 20 kb) Additional file 5:
**Gblock edited alignments in .pdf format.** (ZIP 1181 kb) Additional file 6:
**Gblock edited alignments, truncated for PhyML, .phy files in .txt format.** (ZIP 24 kb) Additional file 7:
**Blastx of** ***W. smithii*** **contigs against** ***D. melanogaster*** **orthologs in the CEGMA database.** (XLSX 116 kb) Additional file 8:
**Contig and singleton annotations.** (XLSX 8403 kb)

## Abbreviations

PTM: Post-translational modifiers
TTFL: Transcription-translation feedback loops
