# Supplementary material for: Evolutionary divergence of core and post-translational circadian clock genes in the pitcher-plant mosquito, Wyeomyia smithii
Source: BMC Genomics. 2015 Oct 6;16:754. doi: 10.1186/s12864-015-1937-y (PMC4594641; doi:10.1186/s12864-015-1937-y)
Supplement: Additional file 2: — Maximum likelihood trees in Newick format; visualize with FigTree [ 116 ]. (DOC 28 kb) [file 12864_2015_1937_MOESM2_ESM.doc]

Additional File 2. Maximum likelihood trees in Newick format. Species abbreviations for gene numbers:

AA, *Aedes aegypti*

AG, *Anopheles gambiae*

CP, *Culex pipiens*

DM, *Drosophila melanogaster*

DP, *Danaus plexippus*

NV, *Nasonia vitripennis*

WSc, *Wyeomyia smithii* contig

WSs, *Wyeomyia smithii* singleton

CKIIA

(DP203677:0.0405273533,NV006496:0.034598339,(DM0264492:0.0604470967,(WSc15735:0.0029418124,(AA012094:1.0E-10,(AG005569:0.0249562165,CP801916:0.0031172287):0.0028305889):0.0030575494):0.0142693602):0.0371544106);

CLK

((CPIJ002146:0.0191835043,AAEL012562:0.0048783403):0.0047835604,((FB0023076:0.4105502051,(Na031986:0.2002790738,DP206046:0.287366244):0.0756565586):0.0654705432,AGAP005711:0.0158853055):0.0219367817,WSs2GK8YT:0.1175354744);

CRY2

(DP208079:0.2563539552,NV006626:0.1812799647,(WSc05165:0.0221351363,((AA002602:0.0898871349,AA011967:0.0000023816)0.9100000000:0.0180802194,(AG004261:0.0492421567,(CP015481:0.0000000001,CP018859:0.0000000001)0.9980000000:0.0350434180)0.3610000000:0.0057495257)0.7200000000:0.0037781877)0.9920000000:0.0873957532);

CULL3

(((((CP801799:0.0134075449,WSc07535:0.0119850173):0.0046644065,(AA007187:1.0E-10,AA006291:1.0E-10):0.0063846566):0.030533083,(AG012905:1.5612E-6,AG008105:2.3572E-6):0.0417927486):0.0763032367,DM0261268:0.2265120849):0.1096228662,NV003449:0.1692048053,DP215829:0.1418020598);

CWO

(((((CP002379:0.0541540348,(AG003844:0.1386476281,AA010513:0.0432596350)0.7870000000:0.0126896724)0.4860000000:0.0165607594,WSc15437:0.0192250286)0.9190000000:0.2254529352,DM0259938:0.4826684258)0.9560000000:0.5328905263,DP209925:1.1656907813)0.9700000000:0.8059957457,NV012954:0.0000040000,NV012953:0.0935453678);

CYC

((((CP014938:0.0138782186,(WSc17314:0.0761326416,(AG005655:5.3333E-6,AG012873:1.0E-10):0.0228499272):0.0101588007):0.0038781393,AA002049:0.0026077665):0.043469787,NV007066:0.1667124116):0.072894693,DM0023094:0.2977714539,DP03011:0.4905737413);

DBT

(CP003503:5.547E-7,(AG008476:1.0590291563,(DM0002413:0.2291009183,(DP200370:0.3337206472,NV018299:0.2399088755):0.0736324957):0.0724039045):0.058557958,(AA002226:0.0667128404,WSc08662:0.0198239592):0.0038769932);

JET

(DP215430:0.8100000631,DM0031652:0.5577772534,(AG009335:0.2195294127,(WSc03197:0.2283849623,(CP802280:0.228393,AA012126:0.1098562225):0.0704570273):0.1418914451):0.2553319923);

KAY

(DP214573:0.6616858708,NV012316:0.743666842,(DM0001297:1.3255618003,(AG001093:0.2666269603,(WSc13013:0.0560219591,(CP003767:0.0620304617,AA008953:0.0998183683):0.0146376559):0.1074302044):0.2085507689):0.2097776444);

NMO

(DP212179:0.4261288217,NV006484:0.0543949859,(DM0011817:0.0264697785,(AG005898:0.0192994375,(WSc17870:0.0308584546,(CP004910:0.0027703385,(AA004797:3.459E-7,AAEL012269:0.0248158041):0.0057247524):5.13289E-5):0.006239122):0.0089900461):0.0294437587);

PDP1

(NV007073:0.3334640548,DP203016:0.1909368631,(DM0016694:0.254826653,(WSc02480:2.0E-10,(CP014920:0.0341534272,(AG006376:0.1554450369,AA005255:0.0348158369):4.555E-7):0.0085434325):0.0947741344):0.2692871466);

PER

(DP203908:0.7869751979,NV013801:1.0260553555,(DM0003068:0.3072604787,(WSs1A331S:0.1971075951,(AG001856:0.0368192656,(CP007193:0.0952130959,AA008141:0.0674552136):0.0303882734):0.0250665177):0.1556474265):0.1187981966);

PP2A-B’

(CP014701:0.6145199264,CP014698:0.2978127576,(CP019419:4.0E-10,((DM0042693:0.0916657359,(NV002993:0.0940893107,DP214593:0.0957512306):0.0460302704):0.0791924368,(AA014031:0.0132399739,(WSc04554:0.0091686741,AG009341:0.0839964254):0.0103856894):2.969548E-4):0.011255457):0.0143483382);

SGG

(NV027316:0.198698555,DP206625:0.1629518347,(DM0003371:0.1499787854,(AG004443:0.0509110379,(CP006114:0.0232220306,(WSc17080:0.0242092708,AA005238:0.0181848288):0.029081864):0.0438731276):0.041635724):0.1019169248);

SLIMB

(DP201583:0.191729012,NV001694:0.1347498389,(DM0023423:0.1259403098,((CP017907:2.0E-10,(WSc03120:0.0101124783,(AA003371:0.0060512629,AG001944:0.0414912249):1.0E-10):0.0020043487):3.35E-7,CP017102:3.194E-7):0.0567214584):0.059573583);

TIM

((((CP801582:0.0920908231,WSc06527:0.1111628049):0.0085230139,AA006411:0.0832549324):0.034480556,AG008288:0.0362864591):0.1603890402,DM0014396:0.2757888797,DP214179:0.5907186172);

VRI

((NV001221:0.7122900142,DP208606:0.4570409347):0.1550087208,(((CP016941:0.0219257258,AA011371:0.0565860458):0.0106077087,AG007801:0.3033275898):0.0055925361,WSc08372:0.0293289963):0.2990204079,DM0016076:0.8252033101);
