# Supplementary figures and images for: Evolutionary divergence of core and post-translational circadian clock genes in the pitcher-plant mosquito, Wyeomyia smithii
Source: BMC Genomics. 2015 Oct 6;16:754. doi: 10.1186/s12864-015-1937-y (PMC4594641; doi:10.1186/s12864-015-1937-y)

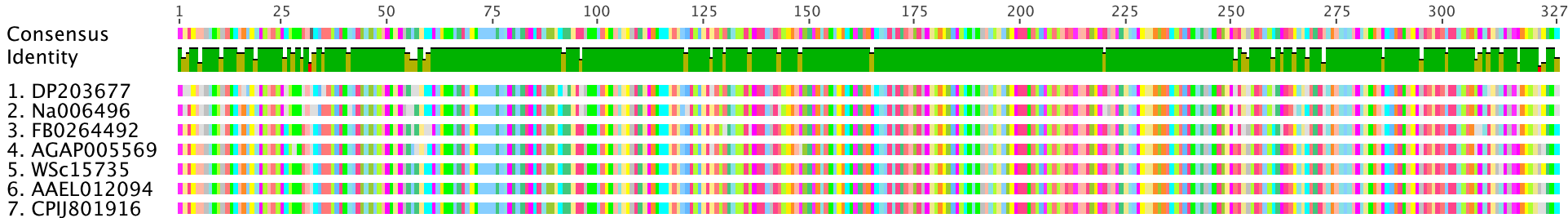

Supplement: Additional file 5: — Gblock edited alignments in .pdf format. (ZIP 1181 kb) [file 12864_2015_1937_MOESM5_ESM.zip › Additional File 7 BGlock Edited files in pdf format/CKIIA_Muscle_Protein_Alignment_RA-gb-1.pdf]

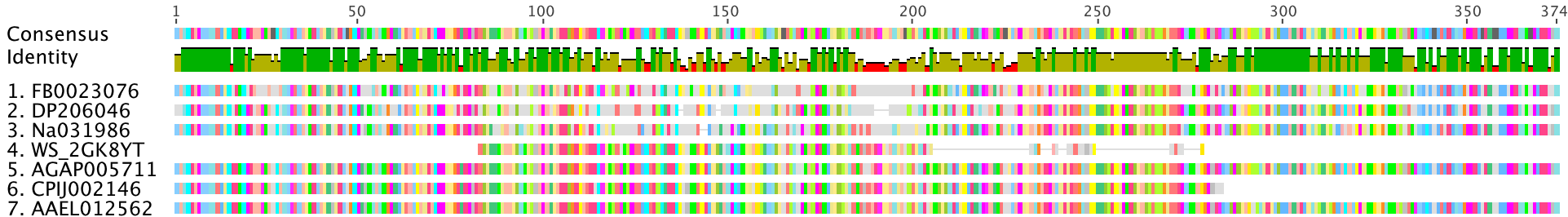

Supplement: Additional file 5: — Gblock edited alignments in .pdf format. (ZIP 1181 kb) [file 12864_2015_1937_MOESM5_ESM.zip › Additional File 7 BGlock Edited files in pdf format/CLK_Muscle_Protein_Alignment_RA-gb-1.pdf]

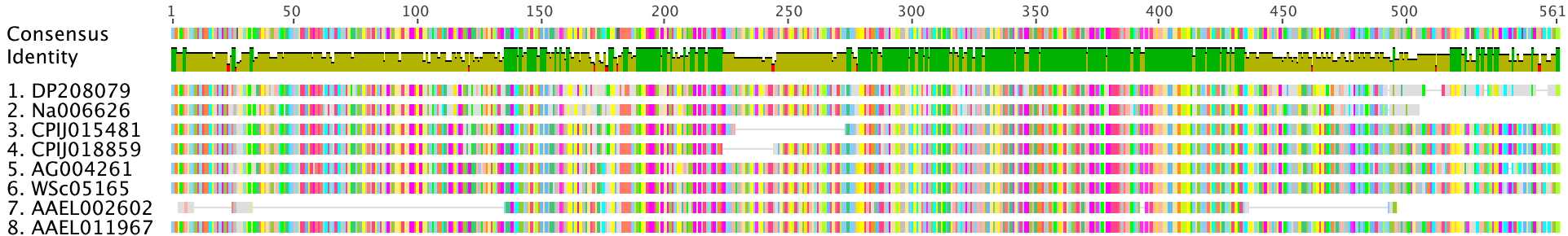

Supplement: Additional file 5: — Gblock edited alignments in .pdf format. (ZIP 1181 kb) [file 12864_2015_1937_MOESM5_ESM.zip › Additional File 7 BGlock Edited files in pdf format/CRY2_Muscle_Protein_Alignment_RA-gb-1.pdf]

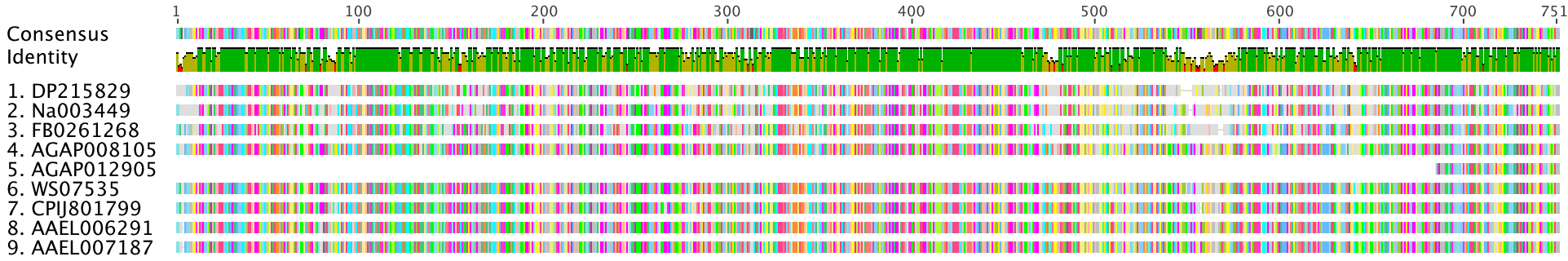

Supplement: Additional file 5: — Gblock edited alignments in .pdf format. (ZIP 1181 kb) [file 12864_2015_1937_MOESM5_ESM.zip › Additional File 7 BGlock Edited files in pdf format/CULL-3_Muscle_Protein_Alignment_RA-gb-1.pdf]

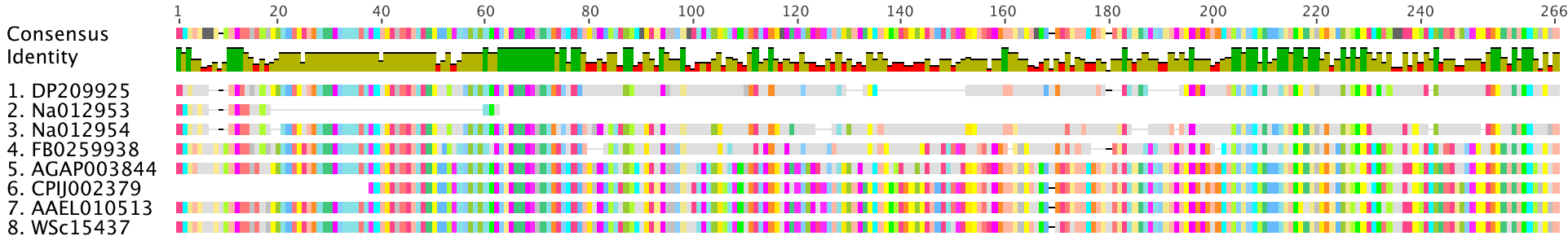

Supplement: Additional file 5: — Gblock edited alignments in .pdf format. (ZIP 1181 kb) [file 12864_2015_1937_MOESM5_ESM.zip › Additional File 7 BGlock Edited files in pdf format/CWO_Muscle_Protein_Alignment_RA-gb.pdf]

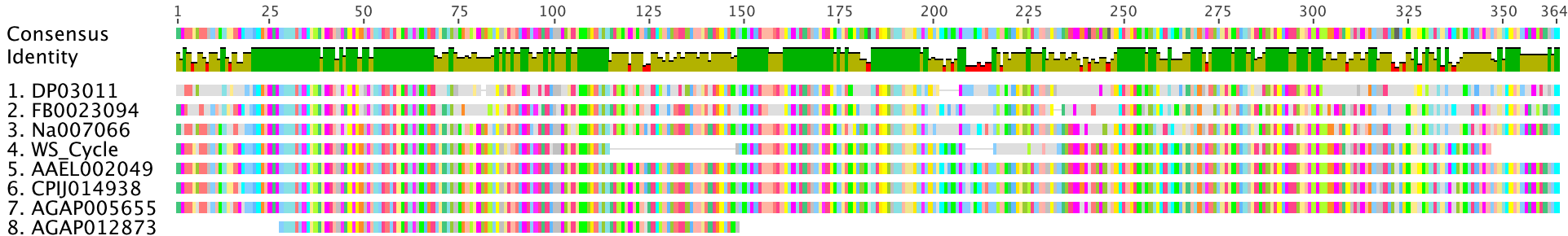

Supplement: Additional file 5: — Gblock edited alignments in .pdf format. (ZIP 1181 kb) [file 12864_2015_1937_MOESM5_ESM.zip › Additional File 7 BGlock Edited files in pdf format/CYC_Muscle_Protein_Alignment_RA-gb-1.pdf]

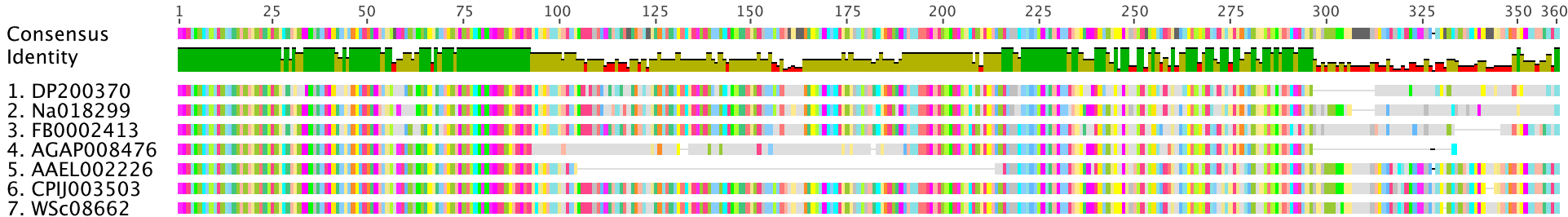

Supplement: Additional file 5: — Gblock edited alignments in .pdf format. (ZIP 1181 kb) [file 12864_2015_1937_MOESM5_ESM.zip › Additional File 7 BGlock Edited files in pdf format/DBT_Muscle_Protein_Alignment_RA-gb-1.pdf]

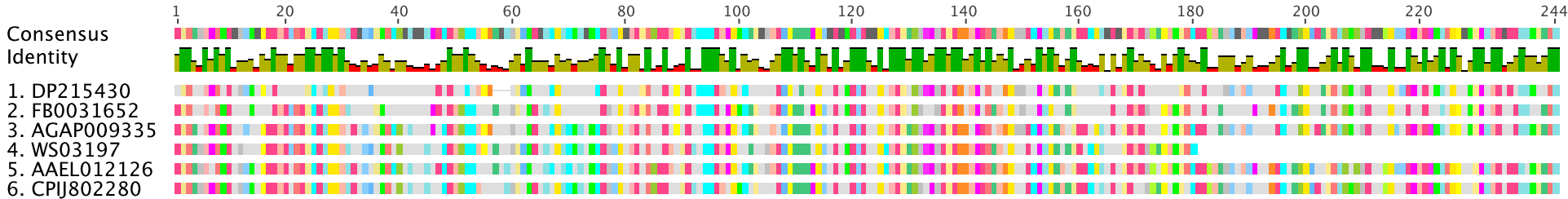

Supplement: Additional file 5: — Gblock edited alignments in .pdf format. (ZIP 1181 kb) [file 12864_2015_1937_MOESM5_ESM.zip › Additional File 7 BGlock Edited files in pdf format/Jetlag_Muscle_Protein_Alignment_RA-gb-1.pdf]

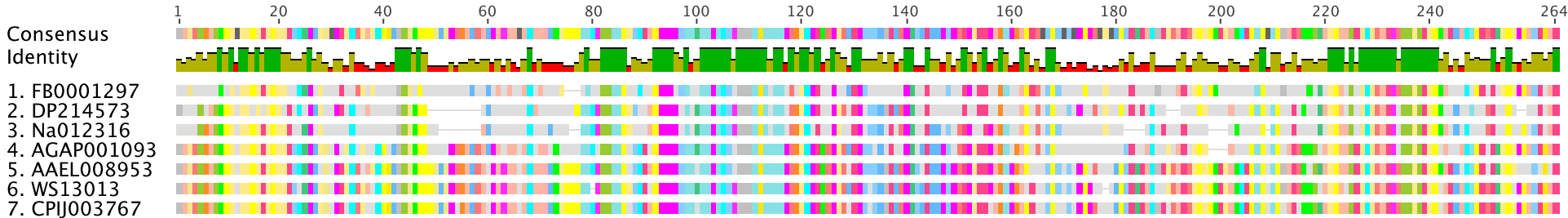

Supplement: Additional file 5: — Gblock edited alignments in .pdf format. (ZIP 1181 kb) [file 12864_2015_1937_MOESM5_ESM.zip › Additional File 7 BGlock Edited files in pdf format/KAY_Muscle_Protein_Alignment_RA-gb.pdf]

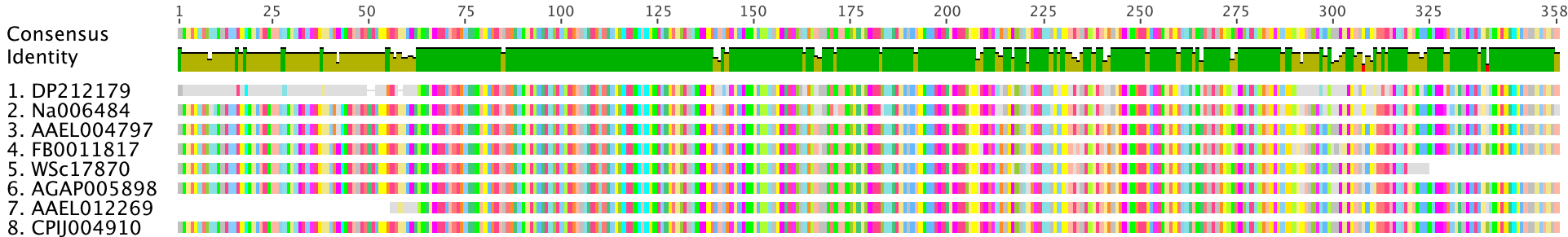

Supplement: Additional file 5: — Gblock edited alignments in .pdf format. (ZIP 1181 kb) [file 12864_2015_1937_MOESM5_ESM.zip › Additional File 7 BGlock Edited files in pdf format/NMO_Muscle_Protein_Alignment_RA-gb.pdf]

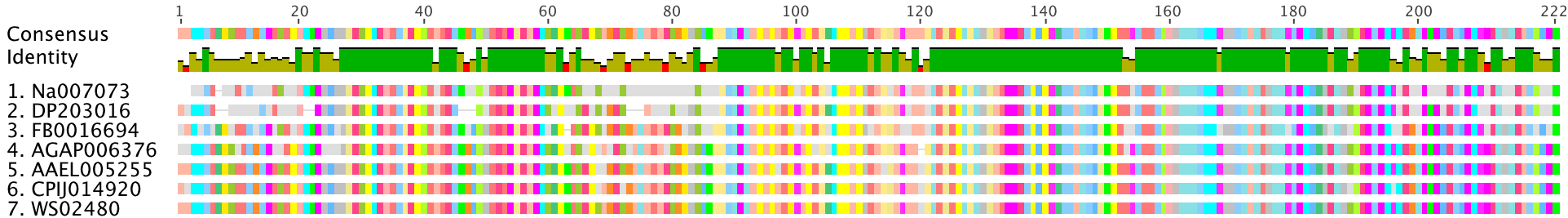

Supplement: Additional file 5: — Gblock edited alignments in .pdf format. (ZIP 1181 kb) [file 12864_2015_1937_MOESM5_ESM.zip › Additional File 7 BGlock Edited files in pdf format/PDP1_Muscle_Protein_Alignment_RA-gb.pdf]

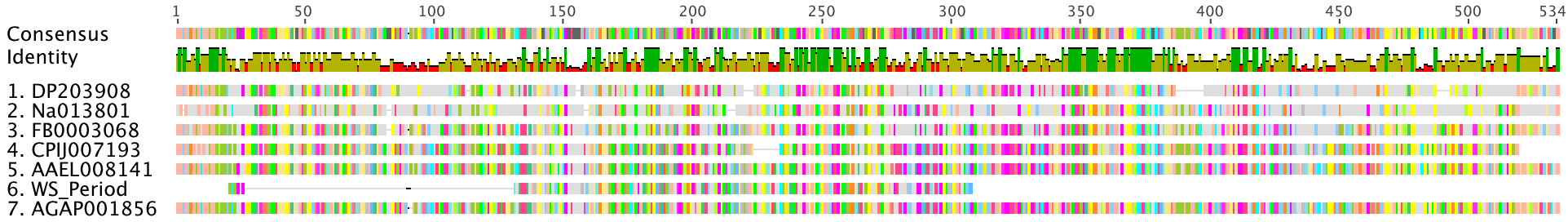

Supplement: Additional file 5: — Gblock edited alignments in .pdf format. (ZIP 1181 kb) [file 12864_2015_1937_MOESM5_ESM.zip › Additional File 7 BGlock Edited files in pdf format/PER_Muscle_Protein_Alignment_RA-gb.pdf]

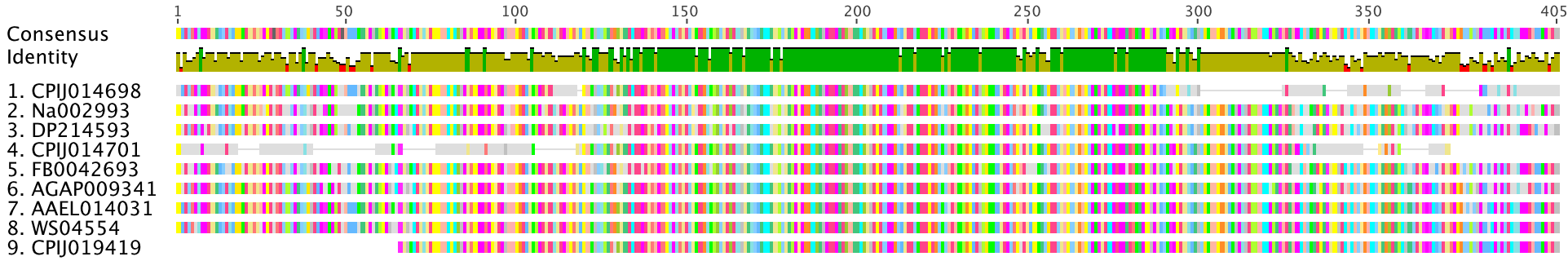

Supplement: Additional file 5: — Gblock edited alignments in .pdf format. (ZIP 1181 kb) [file 12864_2015_1937_MOESM5_ESM.zip › Additional File 7 BGlock Edited files in pdf format/PP2A-B_Muscle_Protein_Alignment_RA-gb-1.pdf]

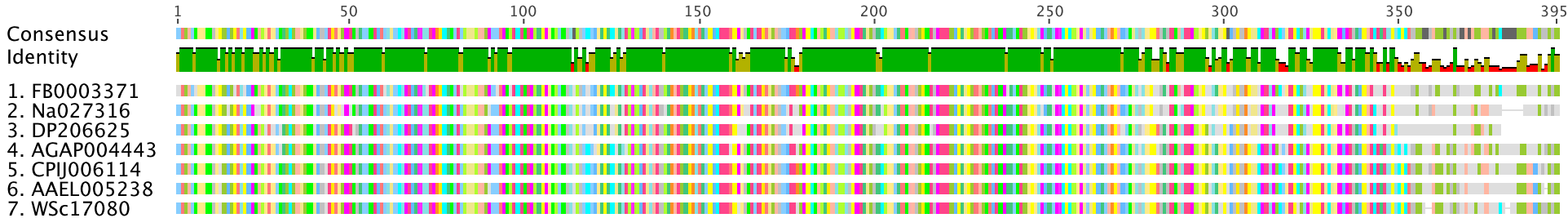

Supplement: Additional file 5: — Gblock edited alignments in .pdf format. (ZIP 1181 kb) [file 12864_2015_1937_MOESM5_ESM.zip › Additional File 7 BGlock Edited files in pdf format/SGG_Muscle_Protein_Alignment_RA-gb-1.pdf]

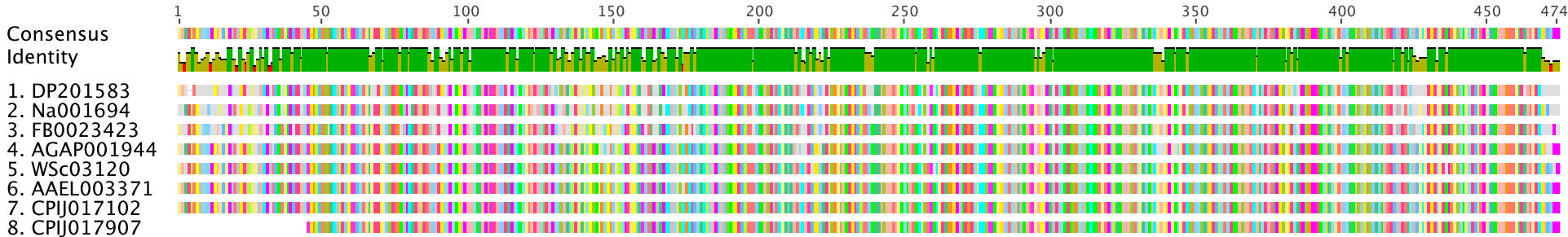

Supplement: Additional file 5: — Gblock edited alignments in .pdf format. (ZIP 1181 kb) [file 12864_2015_1937_MOESM5_ESM.zip › Additional File 7 BGlock Edited files in pdf format/SLMB_Muscle_Protein_Alignment_RA-gb.pdf]

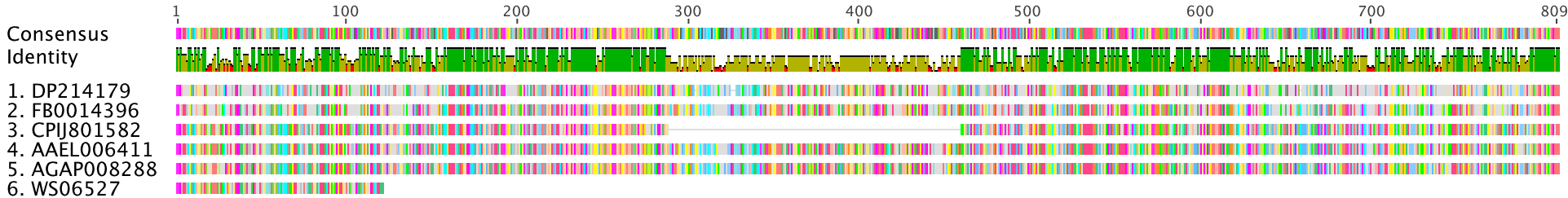

Supplement: Additional file 5: — Gblock edited alignments in .pdf format. (ZIP 1181 kb) [file 12864_2015_1937_MOESM5_ESM.zip › Additional File 7 BGlock Edited files in pdf format/TIM_Muscle_Protein_Alignment_RA-gb-1.pdf]

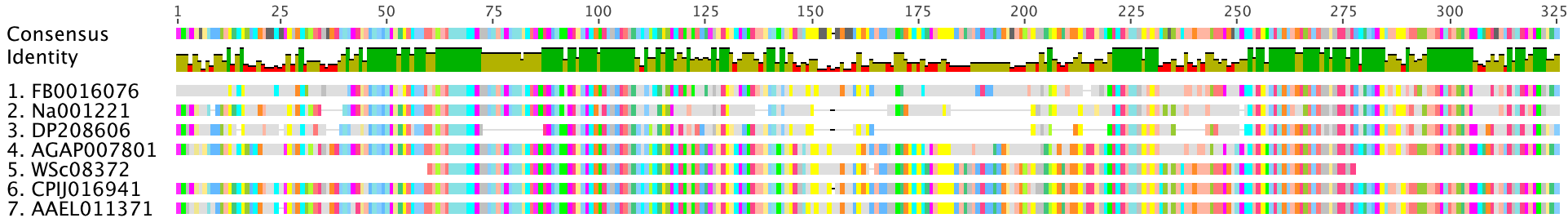

Supplement: Additional file 5: — Gblock edited alignments in .pdf format. (ZIP 1181 kb) [file 12864_2015_1937_MOESM5_ESM.zip › Additional File 7 BGlock Edited files in pdf format/VRI_Muscle_Protein_Alignment_RA-gb.pdf]
